# Interaction between N6-methyladenosine (m^6^A) modification and toxicant-related neurodegeneration: From neural development to pathophysiology

**DOI:** 10.1016/j.gendis.2025.101984

**Published:** 2025-12-17

**Authors:** Zhou She, Peng Huang, Senlin Luo, Lu Zhang, Hong Peng, Yufen Tang, Yuqiong Chen, Jinwen Luo, Wangxin Duan, Lingjuan Liu, Liqun Liu

**Affiliations:** a Department of Pediatrics, The Second Xiangya Hospital of Central South University, Changsha, Hunan 410011, China; b Department of Pediatric Neurology, Children’s Medical Center, The Second Xiangya Hospital of Central South University, Changsha, Hunan 410011, China; c Hunan Provincial Children’s Developmental and Behavioral Clinical Research Center, Changsha, Hunan 410011, China

**Keywords:** m^6^A modification, Neural stem cell, Neurodevelopment, Neuron, Toxicant-related neurodegeneration

## Abstract

N6-methyladenosine (m^6^A) modification is a crucial epigenetic mechanism that is widely expressed across various tissues and biological systems. It regulates gene expression by influencing the stability and translation of messenger RNAs, thereby affecting key physiological processes such as cell division, proliferation, and apoptosis. m^6^A modification plays an essential role in maintaining normal physiological functions and in the pathogenesis of a variety of diseases. Recent studies have highlighted the involvement of m^6^A in the development of the nervous system and its contribution to neurodegenerative diseases, including Alzheimer’s and Parkinson’s disease. However, research on the involvement of m^6^A in nervous system disorders induced by toxic substances remains limited. This review provides an updated overview of the role of m^6^A in neural development, with a particular focus on exploring the potential mechanisms by which m^6^A contributes to toxicant-related neurodegeneration diseases.

## Introduction

Epigenetic modification refers to modifying genetic material and histones without alterations in DNA sequence, which in turn affects the levels of targeted genes *in vivo* by changing the stability and expression degree of genetic material.^1^ There are a variety of types of epigenetic modification, like histone modification, DNA methylation, chromatin remodeling and RNA methylation. Among these, N6-methyladenosine (m^6^A) modification is a widespread intracellular modification mode, occurring on long noncoding RNAs, messenger RNAs (mRNAs) and micro RNAs. m^6^A mainly affects the transport, splicing, translation and stability of mRNAs, and plays a crucial role in cellular processes such as cell proliferation, differentiation and embryonic development.^2^, ^3^, ^4^ The reversible regulation of m^6^A modification is mediated by the dynamic coordination of methyltransferases, demethylases, and m^6^A-binding proteins, which have been shown to participate in the progression of pathological processes, including tumorigenesis and inflammation.^5^

Among various organs, the central nervous system (CNS) exhibits the highest level of m^6^A deposition, which fluctuates across different developmental stages and adapts to functional demands, highlighting its significant role in the nervous system.^6^^,^^7^ Single-cell sequencing has demonstrated that while m^6^A modification sites across various regions of the brain are highly similar, they also exhibit unique features specific to each region. These modifications are closely linked to neuronal activity and the inflammatory responses of glial cells.^8^, ^9^, ^10^ Additionally, the levels of m^6^A modifications in the brain are affected by factors such as age and disease status. Studies have indicated that abnormal m^6^A modification is closely linked to the onset and progression of neurological diseases such as Alzheimer’s disease (AD), Parkinson’s disease (PD), mood disorders and glioma.^11^^,^^12^ A deeper understanding of the role of m^6^A in brain development could enhance our comprehension of neurological diseases and provide new insights and a theoretical foundation for their diagnosis and treatment.

Environmental toxins are pervasive and pose significant health risks through occupational exposure and maternal–fetal transmission. Certain chemicals can cross the blood–brain barrier, causing neurotoxicity and contributing to neurodegenerative diseases like AD and PD. The mechanisms underlying nervous system damage caused by environmental metalloids or toxic substances are complex, and emerging research suggests a critical role for m^6^A in toxicant-related neurodegeneration.^13^ Diseases of the nervous system caused by environmental toxins often share symptoms similar to neurodegenerative diseases. This review first provides an overview of the role of m^6^A in nervous system development and neurodegenerative diseases before exploring the mechanisms by which m^6^A contributes to toxicant-induced neurodegeneration. Ultimately, this review aims to offer a scientific foundation for identifying new directions in the diagnosis and treatment of these conditions.

## Mechanism of m^6^A modification

**Figure 1:**
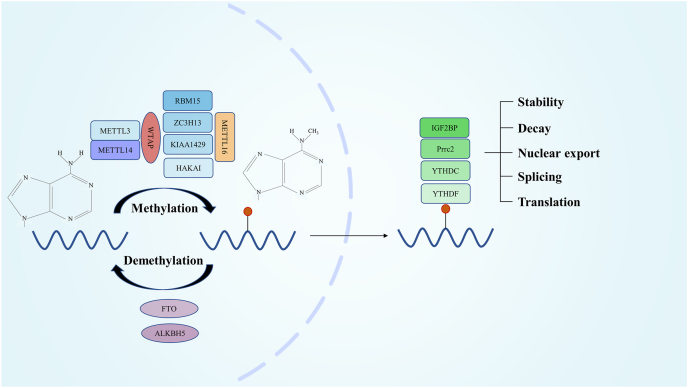
The mechanism of m^6^A RNA methylation. mRNA N6-methylation and demethylation are maintained by “writers” and “erasers”, while “readers” recognize m^6^A sites and perform corresponding regulatory functions.

m^6^A modification is a widespread form of methylation modification in eukaryotic mRNAs. It primarily occurs within the RRACH sequence (R = A or G, H = A, U, or C), which is enriched in long exons, near stop codons, and in the 3′ untranslated regions.^14^^,^^15^ The modification of m^6^A mainly depends on three classes of proteins. The first class is the methyltransferase complex, also known as the “writer”, which includes METTL3, METTL14, and WATP. METTL3 and METTL14 are highly conserved in mammals and function as dimers, with METTL14 recruiting and binding to mRNA, while METTL3 catalyzes the transfer of a methyl group to the N6-adenosine site.^16^^,^^17^ The second class consists of m^6^A-binding proteins, or “readers”, including YTH-domain family proteins 1/2/3 (YTHDF1/2/3) and YTH-domain protein 1/2 (YTHDC1/2), which can recognize and bind to the m^6^A site, and recruit downstream proteins, thus affecting mRNA stability and translational efficiency. Among them, YTHDF1 promotes translation and YTHDF2 induces degradation.^18^, ^19^, ^20^ The third category is m^6^A demethylases, also referred to as “erasers”, which include the fat mass and obesity-associated protein (FTO) and the α-Ketoglutarate-dependent dioxygenase ALKBH5. These proteins are responsible for the demethylation of mRNAs^21^^,^^22^ (Fig. 1).

Currently, m6A detection methods fall into three main categories: global detection, transcriptome-wide high-throughput detection, and single-gene site detection. These techniques allow for the analysis of m6A modification levels under various conditions and their roles in specific diseases or physiological states.^23^ With recent advances, single-cell RNA sequencing has been applied for m6A detection, providing insights into cell-specific and microenvironment-dependent regulation.^24^^,^^25^ In the nervous system, distinct and dynamic m6A modification patterns have been observed across different brain regions and cell states, reflecting the complexity and precision of m6A regulation.^8^^,^^10^

## m^6^A modification and neurodevelopment

Brain development is a process that is precisely regulated in both space and time, and m^6^A modification plays a crucial role in this regulation.^6^^,^^26^ Some researchers have noted that m^6^A modification is controlled with great precision and is involved in the development of the postnatal mouse cerebellum. The targeted inactivation of METTL3 in the nervous system of mice leads to severe defects in brain development. Additionally, mice with METTL3 knockout exhibit significant reductions in both brain size and cerebral cortex volume,^27^ a similar effect observed in mice with FTO knockout.^28^ METTL3-mediated m^6^A modification also contributes to cerebellar development by influencing the mRNA stability of genes related to apoptosis and cerebellar growth.^29^ Under conditions of hypobaric hypoxia, Alkbh5-deficient mice display imbalanced levels of m^6^A modification in the cerebellum. This imbalance results in increased extracellular RNA excretion efficiency and significant alterations in cerebellar phenotypes, including disorganized neuronal structures and abnormal cell proliferation and differentiation. Furthermore, in the cerebellum with disrupted gene expression, Purkinje cells are unable to carry out normal physiological functions and exhibit altered cellular morphology and arrangement.^30^ The brain or cerebellum weight of the mice with abnormal expression of m^6^A-related proteins was markedly lighter than those of the control group, resulting in an inability to maintain normal physiological functions. In addition, YTHDF1, YTHDF2 and WTAP can influence the synaptic connections between Purkinje cells and granule cells. YTHDF1 and YTHDF2 can control the axon growth of granule cells and the development of parallel fibers. In knockout models lacking YTHDF1 and YTHDF2, improved synaptic connections between parallel fibers and Purkinje cells were observed, along with increased locomotor activity in the mice.^31^ In contrast, the knockout of WTAP led to extensive degeneration and necrosis of Purkinje cells, resulting in early-onset ataxia and cerebellar atrophy.^32^

**Figure 2:**
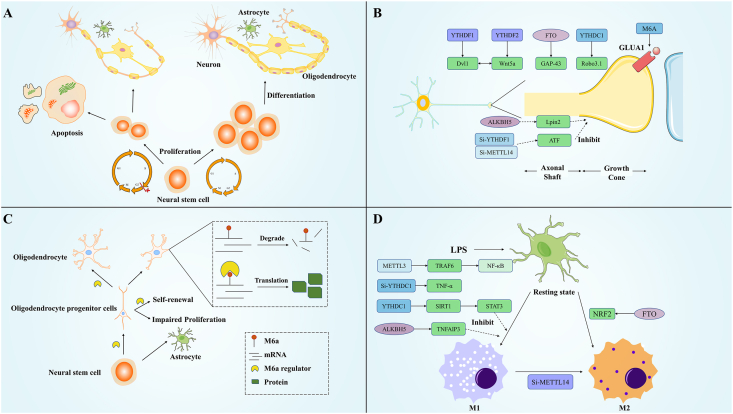
m^6^A and neurodevelopment. **(A)** Under normal circumstances, NSCs multiply and differentiate to produce various nerve cells, which form a neural network. When m^6^A is abnormal, the cell cycle of NSCs arrests, and some NSCs decreases through apoptosis, which leads to a decrease in the number of NSC. The differentiated nerve cells were abnormal. **(B)** In growing axons, some m^6^A proteins inhibit axon growth, while others promote it. **(C)** Normally, NSC outcomes differentiate toward OPCs, which subsequently give rise to oligodendrocytes and myelin formation. A lack of some m^6^A enzymes (e.g., METTL3, 14, prrc2) results in NSC differentiation toward astrocytes, decreased OPC self-renewal capacity, decreased mRNA stability of myelin-forming related proteins (e.g., Olig2, MAG) in oligodendrocytes, and the inability to generate myelin. **(D)** Microglia are regulated by different proteins to differentiate towards the M1 and M2 types.

Except its role in brain development, m^6^A is also essential for processes such as neuronal stem cell (NSC) differentiation, neuronal migration, and synapse formation. For example, m^6^A modification regulates the differentiation of NSCs into neurons by controlling the expression of key genes that drive neuronal generation. Additionally, m^6^A modification is vital for synapse formation, modulating the translation and stability of synaptic proteins, which ultimately influences both the quantity and quality of synapses (Table 1 and Fig. 2).

**Table 1:** m^6^A and neurodevelopment.

m^6^A	Protein	Target	Function	References
Writer	Wtap		Purkinje cell degeneration and necrosis, cerebellar atrophy, ataxia	32
	METTL3, 14, WATP	Tyrobp	Participate in learning, cognitive function	85
	METTL3, 14	Lrp24	Promote neuron proliferation and differentiation, and destroy hippocampal function	38
	METTL3, 14		Deletion prolongs the cell cycle of NSC and affects its function	35
	METTL3		Participate in TRAF6-NF-κB pathway to ensure microglia cell activation	59
	METTL3	Ezh2	Absence leads to cell cycle disorder, inhibits the proliferation of aNSCs and affects neuronal development and favors glial cell lines	36
	METTL3		Hippocampal neuron degeneration, apoptosis, memory function impaired	37
	METTL3	STUB1	Enhances p-Tau clearance	86
	METTL3		Positively correlated with fear memory discrimination	77
	METTL3		Promote long-term memory	75
	METTL3		Affects brain development	27
	METTL3		Affects granule cells and cerebellar development	29
	METTL3		Affects purkinje cell development	30
	METTL14	Tyrosine hydroxylase	Decreased tyrosine hydroxylase and impaired motor function	70
	METTL14		Affect the proliferation and differentiation of NSC	39
	METTL14	Arhgef2	Interfere the expression of Npdc1 and Cend1 and ensure the normal development of neurons	46
	METTL14	ATF3	Promote posterior axon repair of SNL	120
	METTL14	MBP, MAG	Myelin development	54
Eraser	FTO	–	Absence inhibit the production and development of nerve cells	43
	FTO		Absence reduces the number of proliferative NSCs, affects brain development and impaired cognitive and learning functions	28
	FTO	NMDAR1	Increases oxidative stress and calcium influx, leading to the death of dopamine neurons	69
	FTO		Affects dopamine receptor activity	68
	FTO	GAP43	Regulates axon growth	47
	FTO		Knockout solidifies fear memories	76
	FTO		Inhibits memory formation	80
	ALBKH5	A20	Inhibits microglia M1 polarization	61
	ALBKH5		Effect granulogenesis	30
	ALBKH5	Dgkh	Reduces tau protein phosphorylation	87
	ALBKH5	Lpin2	Inhibits axon growth	121
Reader	YTHDF1, 2	Dvl1, Wnt5a	InhibitS the growth of cerebellar parallel fibers and granulosa cell axons *in vivo*	31
	YTHDF2		Inhibits the differentiation of iPSCs into nerve cells	42
	YTHDF2		Knockout results in early embryo death, abnormal cortical development, and decreased basal progenitor cells	40
	YTHDF2		Ensures the normal growth of granule cells MF	84
	YTHDF1, 3		Ensures normal synaptic transmission	122
	YTHDF1	Robo3.1	Guides axon growth	48
	YTHDF1		Necessary for short-term memory formation	79
	YTHDF1		Promotes learning and long-term memory	78
	YTHDC	Sirtuin 1	Inhibits microglia M1 polarization	60
	Prrc2a	Olig2	Oligodendrocytes produce and develop normally	55
	Igf2np	TNF-α	Knockout inhibits TNF-α production	58

### m^6^A modification and NSCs

Neural stem cells (NSCs), also known as radial glial cells (RGCs), play a vital role in the nervous system. In early-stage embryos, NSCs rapidly expand through continuous mitosis. During the middle and late stages of embryonic development, they undergo asymmetric division, which generates neural precursor cells and glioblasts. These precursor cells eventually develop into neurons and astrocytes or oligodendrocytes. A certain number of undifferentiated NSCs are maintained in the subventricular region of the lateral ventricle and the subgranular zone of the dentate gyrus.^33^^,^^34^ The processes of division and differentiation of NSCs are precisely regulated and remain in an active state. The persistent expression of a significant amount of DNA is continuously maintained, and influenced by various epigenetic regulations. Among these, m^6^A modification has emerged as particularly significant.^6^^,^^14^ Previous studies have indicated that m^6^A modification may influence both the self-renewal and differentiation of NSCs.

It was found that the S-G2-M phases of the cell cycle in RGCs were significantly prolonged following the knockout of METTL14 in the embryonic mouse brain. This disruption reduced the efficiency of cell division by slowing down DNA replication and chromosome formation. Similar results were observed with the knockout of METTL3, which blocked the differentiation of RGCs into downstream cells.^35^ In adult neural stem cells (aNSCs), the absence of METTL3 caused most cells to arrest in the S phase, leading to a decreased percentage of cells in the G2 and M phases. This suggests a reduction in their ability to divide. Gene enrichment analysis revealed that the methylation levels of mRNAs related to the cell cycle and stem cell differentiation decreased when METTL3 and METTL14 were absent. This reduction in methylation could result in an inability to identify and clear mRNA in time, impacting the cell cycle.^36^ METTL3 deficiency has been associated with disruptions in the cell cycle and may result in a reduction in stem cell populations through the induction of apoptosis.^37^ Additionally, METTL3 deficiency has been linked to disruptions in the cell cycle, which may lead to a decline in stem cell populations due to the induction of apoptosis. The knockdown of either METTL3 or METTL14 significantly reduced the proliferation of NSCs, indicating that both proteins are essential for their division. However, their overexpression exhibited different effects. Specifically, overexpressing METTL3 enhanced cell proliferation and neuronal differentiation, while overexpressing METTL14 only mitigated the negative effects seen after knockdown and did not promote proliferation in normal cells.^38^^,^^39^

The deletion of the writer can impact the function of NSCs, but impairments in the functions of readers and erasers can also lead to abnormalities in NSCs. Research has shown that embryos of mice lacking the YTHDF2 gene do not develop normally and tend to die within a concentrated timeframe of 14.5–18.5 days. Additionally, half of the heterozygous embryos did not survive, primarily due to abnormal development of the nervous system. Anatomical studies of the embryos revealed developmental delays in the cerebral cortex and a reduction in the division of apical precursor cells in homozygous rats. This decline resulted in fewer downstream basal cells, ultimately hindering the development of normal neurons. NSCs extracted from these embryos also demonstrated a diminished capacity for division and were only able to differentiate into neurons that exhibited abnormal morphology, without forming glial cell lineages.^40^ Furthermore, postnatal knockdown of YTHDF2 in mice led to an increase in TGF-β expression in NSCs located in the dentate gyrus. This change caused the cells to enter a quiescent state, impairing their normal ability to proliferate and generate neurons.^41^ In induced pluripotent stem cells, YTHDF2 can inhibit the translation of mRNAs related to the nervous system, preventing their differentiation into nerve cells.^42^ In a study where the FTO gene was knocked down in mice, researchers observed changes in m^6^A modification and the expression of several important components of the brain-derived neurotrophic factor (BDNF) pathway within brain tissue. These alterations impair the self-renewal capacity of aNSCs and their differentiation potential, leading to a decreased number of both neurons and glial cells.^28^ Conversely, in a separate experiment, FTO knockdown in mouse aNSC was associated with enhanced proliferation and neuronal differentiation. However, the differentiated neurons exhibited abnormal development, characterized by a significant reduction in axon length and fewer crossover points.^43^

These studies mentioned indicate that m^6^A-related enzymatic proteins can influence the function of NSCs by prolonging the cell cycle and promoting apoptosis. This can result in abnormalities in the number, morphology, and function of downstream neurons. If m^6^A modifications are disrupted during growth, they could adversely affect the development of the nervous system and lead to neurodevelopmental disorders (Fig. 2A). The literature reports four cases of individuals with abnormal YTHDF3, all of whom exhibit developmental delays and varying degrees of intellectual disability.^44^ Additionally, the Fragile X messenger ribonucleoprotein interacts with YTHDF2 to target numerous mRNAs containing the m^6^A marker, helping to maintain their stability. When this process is dysfunctional, Fragile X syndrome may occur.^45^ Overall, abnormal m^6^A modifications can lead to disorders in nervous system development. Whether it is possible to induce stem cell differentiation through the regulation of m^6^A modification and thereby repair the nervous system requires further investigation.

### m^6^A modification and neurons

Neurons are the basic components of neural networks and signaling pathways. The development of neurons and the proper establishment of synaptic connections are essential for emotions, learning, memory, and actions. As previously mentioned, deficiencies in various m^6^A regulatory proteins can impact the proliferation and differentiation of NSCs, which not only alters the number of neurons but also affects the morphology of differentiated neurons. These changes are primarily characterized by a reduced number of dendrites, shorter axons, and fewer connections with other neurons^36^^,^^43^^,^^46^ (Fig. 2B).

The development of axons and the formation of synapses are fundamental processes that establish connections between neurons, enabling them to transmit information to neighboring cells. In axons, the levels of m^6^A modification on mRNAs are high, and this modification can influence and direct axon growth by altering the expression of specific proteins, such as FTO. When FTO is knocked down, the m^6^A modification of GAP-43 mRNA increases, which is accompanied by reduced translation and inhibited local axon growth. This suggests that FTO regulates axon growth by controlling the m^6^A modification levels of GAP-43 mRNA.^47^ Additionally, during the midline crossing of spinal commissural axons, YTHDF1 recognizes the m^6^A site on the guidance receptor of Robo3.1 mRNA and enhances its translation. Knocking out YTHDF1 specifically in spinal commissural axons leads to defects in axon guidance.^48^ Furthermore, with the assistance of the lincRNA Dubr, the YTHDF1/3 complex promotes the translation of tau and calmodulin, thereby supporting axon growth in neurons.^49^

### m^6^A modification and glial cells

Glial cells, which include astrocytes, oligodendrocytes, and microglia, are essential components of the nervous system. They provide neurons with nourishment, protection, and support. Astrocytes, the most abundant glial cells in the mammalian brain, originate from NSCs in the subventricular zone. Their functions include filling the extracellular matrix, supporting neurons, and forming the blood–brain barrier.^50^ Oligodendrocytes develop from oligodendrocyte progenitor cells (OPCs) that differentiate from NSCs. They play a crucial role in wrapping axons with myelin sheaths, which accelerates the conduction velocity of action potentials. Additionally, oligodendrocytes provide nutritional and metabolic support to axons.^51^^,^^52^ The production of glial cells derived from NSCs is regulated by m^6^A modification. When NSCs are induced to differentiate, the expression of m^6^A modifications influences their differentiation pathway. Multiple experimental studies have shown that the absence of the methyltransferases METTL3 or METTL14 can drive NSCs toward glial cell lineages. However, the lack of METTL14 has also been found to prolong the proliferation of RGCs and hinder their differentiation.^35^^,^^36^^,^^38^ Additionally, a deficiency in FTO decreases the differentiation of stem cells into astrocytes.^43^ Following spinal cord injury, METTL3 and IGF2BP2 collaboratively regulate astrocyte activation. METTL3 facilitates the methylation of YAP1 mRNA, while IGF2BP2 maintains the stability of YAP1, thereby supporting astrocyte function. The knockdown of METTL3 impedes astrocyte proliferation, resulting in substantial infiltration of inflammatory cells, heightened neuronal loss, compromised axonal regeneration, and impaired functional recovery.^53^ Additionally, the regulation of oligodendrocytes is linked to METTL14. Oligodendrocyte precursor cells with METTL14 knockdown do not mature properly, leading to reduced expression of myelin proteins and myelin-related glycoproteins. Consequently, the number of oligodendrocytes decreases, affecting myelin formation in the CNS and slowing the velocity of neuronal conduction.^54^ Prrc2a plays a crucial role in the generation and differentiation of oligodendrocytes. The absence of Prrc2a results in NSCs differentiating into astrocytes instead of OPCs, thereby reducing the OPC population. Moreover, the lack of Prrc2a diminishes the self-renewal capacity of OPCs and impairs their differentiation into oligodendrocytes. Additionally, the absence of Prrc2a affects the stability of myelin-associated transcripts within oligodendrocytes, leading to reduced myelin formation.^55^ It was later found that Prrc2b performs a similar function^41^ (Fig. 2C). Abnormalities in astrocytes and oligodendrocytes can lead to a variety of diseases. For instance, dysfunction in astrocytes can damage the blood–brain barrier, impede microglial migration, and trigger inflammation. Abnormal oligodendrocyte function has been linked to several demyelinating diseases, such as multiple sclerosis.^51^^,^^52^ Regulating m^6^A modification may help repair dysfunctional cells and inhibit tumor growth by controlling the occurrence and function of glial cells.

Microglia cells are derived from macrophages and dendritic cells in the blood. As critical immune cells in the CNS, microglia play a crucial role in the inflammatory response by phagocytosing pathologically folded proteins, such as tau proteins and myelin fragments, thereby inhibiting the progression of inflammation.^52^^,^^56^ Research has shown that the levels of m^6^A modification differ significantly among M0, M1, and M2 microglia cells, suggesting that m^6^A modification is involved in the anti-inflammatory processes of these cells.^57^^,^^58^ In LPS-induced proinflammatory microglia, the levels of METTL3 were found to increase significantly and were positively correlated with the inflammatory protein TRAF6. Overexpression of METTL3 promotes the expression of TRAF6 and NF-κB.^59^ In retinal microglia during uveitis, YTHDC1 helps maintain the stability of SIRT1 mRNA and reduces the phosphorylation levels of STAT3, thus inhibiting the M1 polarization of microglia. Conversely, the absence of YTHDC1 leads to M1 polarization, increased expression of inflammatory factors such as TNF-α and COX2, along with enhanced migration of microglia cells.^60^ In the context of diabetic retinopathy, ALKBH5 regulates the m^6^A modification of TNFAIP3 mRNA, controlling the expression of TNFAIP3 and inhibiting the M1 polarization of microglia.^61^ During ischemic stroke, inhibiting METTL14 has been shown to induce a phenotypic shift in microglia from M1 to M2, which helps attenuate inflammatory damage. In contrast, the up-regulation of FTO has been found to enhance Nrf2 expression in microglia, promoting their polarization toward the M2 type^62^^,^^63^ (Fig. 2D). The inflammatory response involving microglia significantly impacts the stability of CNS. Whether the inflammatory response is intense or absent, it can lead to neurological damage. Further research is needed to investigate whether the immune response of microglia cells can be regulated through m^6^A modification to stabilize the inflammatory response in the nervous system.

## Role of m^6^A modification in neurodegenerative diseases

### Parkinson’s disease

The primary characteristics of PD pathophysiology include the aggregation of misfolded alpha-synuclein (α-Syn) and the depletion of dopaminergic neurons within the substantia nigra.^64^

In cellular and animal models of PD, researchers have observed a marked reduction in m^6^A modification levels alongside an up-regulation of FTO expression. Suppressing FTO has been shown to inhibit α-Syn expression and reduce neuronal apoptosis.^65^ These findings suggest that the increased expression of α-Syn may be related to the decreased m^6^A modification levels in the progression of PD.

Dopaminergic neurons are among the most widely distributed neurons in the brain, and the dopamine they secrete is a crucial neurotransmitter involved in regulating functions such as motor skills and cognition. In a mouse model of PD induced by MPTP, alterations in m^6^A-related proteins have been observed. Specifically, there is an up-regulation of ALKBH5 and IGF2BP2, along with a down-regulation of YTHDF1 and FMR1.^66^ In another MPTP-induced PD mouse model, a significant reduction in METTL3 expression was noted. The down-regulation of METTL3 adversely affects the stability of glutathione reductase, contributing to the degeneration of dopaminergic neurons and subsequent motor dysfunction.^67^ FTO knockout mice exhibit a marked decrease in dopamine neuronal activity, significantly impaired functioning, and reduced locomotor responses to cocaine. However, when FTO is specifically knocked out in dopaminergic neurons, there is an increase in responsiveness to cocaine.^68^ Additionally, research has shown that the presence of FTO results in lower levels of m^6^A modification in PC12 cells, which enhances the expression of N-methyl-d-aspartate receptor 1, leading to increased calcium ion influx and oxidative stress, ultimately worsening cell damage.^69^ Conditional knockout of METTL14 in the substantia nigra results in decreased levels of m^6^A modification in this region, as well as down-regulation of tyrosine hydroxylase, which is essential for dopamine synthesis. Additionally, mice with METTL14 knockdown exhibit impaired motor function and activity.^70^ Knocking down METTL14 in the striatal region alters the expression of synapse-associated proteins, resulting in increased neuronal excitability and long-term impairment of striatal function.^71^ Beyond its impact on neuronal function, m^6^A also contributes to neuronal damage through ferroptosis. In an MPTP-induced PD model, elevated FTO expression reduces Nrf2 mRNA methylation and expression, exacerbating ferroptosis and cellular injury.^72^ Another study identified BRCA1-associated protein 1 (BAP1) as a downstream target of FTO in the same model. FTO decreases BAP1 methylation, preventing its degradation by YTHDF2 and increasing its expression. This FTO-BAP1 axis suppresses SLC7A11 expression, thereby promoting neuronal ferroptosis.^73^ Additionally, studies have shown that overexpressing METTL14 reduces TRAF6 expression, which mitigates mitochondrial damage and reduces the production of reactive oxygen species (ROS) in dopaminergic neurons, ultimately inhibiting ferroptosis.^74^

### Alzheimer’s disease

The hippocampus is a crucial area of the central nervous system involved in learning and memory. m^6^A modification plays a direct role in regulating the physiological functions of neurons within this region. Research has shown that the abundance of m^6^A modification is closely linked to memory enhancement. Specifically, studies indicate that levels of m^6^A modification in the medial prefrontal cortex of mice increase steadily with the accumulation of experiences. When METTL3 is knocked out, the ability to consolidate memory is diminished. However, this decline can be reversed either by intensifying training or by restoring METTL3 expression. Additionally, overexpression of METTL3 enhances the learning and memory abilities of mice. Similarly, overexpression of METTL14 produces comparable effects.^71^^,^^75^^,^^76^ Knocking down METTL3 in the hippocampus leads to various cellular changes associated with AD, resulting in neuronal death, loss of synaptic connectivity, and significant memory deficits alongside cognitive dysfunction in mice.^37^ Additionally, METTL3 levels show a significant positive correlation with the ability to discriminate fear memories.^77^ Moreover, the reading protein YTHDF1 plays a critical role in memory formation. When YTHDF1 is knocked out in mice, their learning and memory functions suffer, as evidenced by the reduced weight of the hippocampus and a significantly longer escape time from a water maze compared to the normal control group. However, re-expressing YTHDF1 can restore the learning and memory functions in these mice.^78^ The main underlying mechanism is that the loss of YTHDF1 impairs basic synaptic transmission in hippocampal neurons and disrupts long-term potentiation. In addition to their impact on long-term memory, both METTL3 and YTHDF1 are essential for the formation of short-term memory in Drosophila mushrooms.^79^ Specific knockout of METTL3 or YTHDF in either neurons or mushroom bodies can cause short-term memory impairment in Drosophila mushrooms. Deletion of the reading protein Prrc2a leads to hypomyelination, impaired motor function and cognitive deficits in mice.^55^ FTO is mainly expressed in the hippocampus dorsal region, which is related to fear emotion and helpful for enhancing the consolidation of fear memory in mice. After experiencing a frightening situation, the expression of FTO in the brains of mice decreases. However, when FTO is knocked out, the memory of fear is strengthened and better consolidated.^76^^,^^80^ Additionally, FTO knockout mice displayed longer escape times in mazes, poorer performance in the eight-arm radial maze, and significant impairments in spatial learning and memory.^28^ Impaired hippocampal function can cause impaired learning and memory function and Alzheimer’s-like symptoms.

The primary clinical manifestation of AD is characterized by deficits in learning and memory, while the principal pathological feature is the accumulation of insoluble neurotoxic aggregates, specifically amyloid-β and tau proteins.^81^ Recent research utilizing sequencing and bioinformatic analysis has demonstrated that m^6^A modification is dynamically regulated during brain development, with a notable increase in m^6^A deposition observed with increasing age. Furthermore, m^6^A modification plays a crucial role in modulating the protein levels of key genes involved in AD-related pathways.^6^^,^^82^ In patients diagnosed with AD, a reduction in m^6^A modification levels has been noted, resulting in decreased production of proteins associated with synaptic function. Additionally, in mouse models of AD, the deletion of YTHDF2 leads to abnormal axonal growth in granule cells, impairing their ability to form typical connections with CA3 neurons.^83^^,^^84^ Some studies have indicated that the inhibition of NSC proliferation in the hippocampus occurs first after knocking out METTL3, leading to a reduction in neuron generation. This results in depression-like behaviors and memory decline in mice. Additionally, decreased levels of m^6^A modification in the hippocampus activate the intracellular oxidative stress response, initiating the apoptosis process and consequently leading to significant neuronal death and cognitive impairment in mice.^38^ The loss of function of the gene encoding casein kinase binding protein induces learning and memory deficits in mice, as well as increased levels of total tau and amyloid beta in both the hippocampus and cerebral cortex. These effects are associated with the down-regulation of METTL3 and METTL14 expression and decreased m^6^A modification levels.^85^ The tau protein is essential for the stability of neuronal microtubules, and its hyperphosphorylation and accumulation are characteristic features of AD. Increased expression of METTL3 promotes autophagy, which results in reduced levels of the tau protein, while the overexpression of ALKBH5 similarly reduces excessive phosphorylation.^86^^,^^87^ In the context of diabetes, FTO activates Tau phosphorylation in an mTOR-dependent manner.^88^ In conclusion, m^6^A modification plays a crucial regulatory role in the hippocampus, which may be involved in the occurrence and progression of AD.

### Epilepsy

Initial investigations revealed a reduction in FTO levels during seizures, with sodium valproate shown to mitigate seizures by enhancing FTO expression in the hippocampus.^89^ In a murine model of epilepsy, a decrease in FTO expression was accompanied by an increase in YTHDF2 expression. The overexpression of FTO reduced the m^6^A methylation levels of Nrf2 mRNA, leading to improved cell viability, inhibited apoptosis, and slowed the progression of epilepsy. Conversely, YTHDF2 binds to the Nrf2 m^6^A locus, leading to decreased Nrf2 expression and promoting the development of epilepsy.^90^ Conversely, YTHDF1 offers a protective effect against the progression of epilepsy. It activates PTEN expression, which inhibits the release of astrocytes and pro-inflammatory cytokines, thereby reducing neuroinflammation and neuronal death.^91^ Similarly, YTHDC2 targets astrocytes and enhances the expression of the glutamate/cystine reverse transporter, which significantly lowers extracellular glutamate levels and decreases seizure activity.^92^ In both murine and cellular models of epilepsy, METTL3 was observed to down-regulate the expression of the vimentin waveform protein, mitigate the inflammatory response, decrease neuronal apoptosis, and delay the progression of seizures.^93^ Following seizure events, WTAP facilitated m^6^A modification, which increases the expression and stability of transient receptor potential visfatin 1. This process helped reduce intracellular ROS, restored mitochondrial function, and subsequently reduced neuronal apoptosis.^94^ The pathogenesis of epilepsy is intricate, and m^6^A modification appears to be a potential therapeutic target; however, further research is required to elucidate its role.

### Toxicant-related neurodegeneration

Various toxic substances are major causes of nervous system damage, leading to neuronal injury and cognitive dysfunction. Among the multiple mechanisms of neurotoxicity, m^6^A modification represents a novel and critical pathway.^95^ Understanding this mechanism is important for preventing and treating related diseases (Table 2).

**Table 2:** Roles of m^6^A in Toxicant-related neurodegeneration.

Toxic substances	Subjects	m^6^A modification	types of study	Mechanisms	References
Sevoflurane	Hippocampus	ALBKH5 up	*In vivo*	Damages neurons and cause cognitive impairment	96
	Hippocampus	YTHDF1 down	both	Causes neuroinflammation, neuronal injury, apoptosis through CREB-BDNF pathway	97,98
	Prefrontal cortex				
	SH-SY5Y cells	METTL3 down	*In vitro*	Impairs neuronal function through METTL3-SOX2 axsis	99
Manganese	Hippocampal	Fto down	*In vivo*	Inhibits SOX2-FTO- NMDA pathway to impaire synaptic function	100
	Striatum	Fto down	*In vitro*	Decrease Ephrin-B2 expression in dopaminergic neurons which disrupting axonal projections and inducing motor dysfunction	101
	Microglia	METTL3 up	both	Promotes the release of inflammatory factors through YTHDF2/IGF2BP axis	102
Arsenite	Cortex	Fto down	both	Leads to a reduction in dopamine neurons	103,104
Aluminum	Hippocampal	METTL3/FTO down	*In vitro*	Reduce BNDF protein expression, thereby inducing oxidative stress and apoptosis	106,107
Cobalt	H4 cells	FTO down	*In vitro*	Results in the overproduction of ROS, G1/S cell cycle arrest, and the upregulation of cleaved caspase 1/3/9, thereby accelerating apoptosis	108
	H4 cells	FTO down	*both*	Induces cell autophagy through increasing the methylation of TSC1-TSC2 complex	109
	H4 cells	ALBKH5 down	*In vitro*	Exacerbates ferroptosis through the regulation of heme oxygenase-1	110,111

### Sevoflurane

Sevoflurane is a commonly used anesthetic that significantly contributes to postoperative cognitive dysfunction by causing neuronal damage and cognitive impairment. Research indicates that repeated exposure to sevoflurane alters the expression of m^6^A regulators in the hippocampus, notably increasing the levels of ALKBH5 and YTH family proteins. The inhibition of ALKBH5 using IXO1 has been shown to substantially reduce the neuronal injury and cognitive deficits induced by sevoflurane.^96^ Conversely, other studies have demonstrated that sevoflurane down-regulates the expression of YTHDF1 in both the hippocampus and prefrontal cortex. Overexpressing YTHDF1 has been found to alleviate neuroinflammation, neuronal injury, apoptosis, and cognitive impairment by modulating Synaptophysin and the cAMP response element binding protein-BNDF (CREB-BDNF) pathway.^97^^,^^98^ Additionally, methylated proteins are involved in sevoflurane-induced cognitive deficits, as evidenced by the down-regulation of METTL3 following exposure, which impairs neuronal function. Activating the METTL3-SOX2 (Sex-determining region Y-box-2) axis has been shown to significantly enhance recovery.^99^

### Manganese

Manganese (Mn) is an essential trace element; however, chronic exposure to it can lead to neurotoxicity and symptoms similar to those of PD. Studies in mice have shown that exposure to Mn results in a decreased expression of FTO in hippocampal neurons. Interestingly, enhancing FTO expression can alleviate the learning and memory deficits caused by Mn exposure. Mn exposure results in decreased phosphorylation of SOX2, which suppresses FTO transcription. This down-regulation in turn lowers the expression of N-methyl-d-aspartate receptors, impairing synaptic function.^100^ Similarly, Mn exposure leads to a decrease in FTO levels in the striatum, resulting in reduced expression of Ephrin-B2 in nigrostriatal dopaminergic neurons. This disruption affects axonal projections and contributes to motor dysfunction.^101^ Additionally, chronic Mn exposure can trigger the overactivation of METTL3 in microglia, which promotes the release of inflammatory factors through the downstream YTHDF2/IGF2BP signaling pathway.^102^

### Arsenite

Arsenite (As) is a toxic metalloid pollutant that accumulates in the CNS, contributing to the development of neurodegenerative diseases. Chronic exposure to As has been shown to impair cognitive function, learning, and emotional well-being in mouse models. Research indicates that As exposure reduces the number of dopamine neurons in the cortex and negatively affects their function, which is linked to a decrease in the expression of FTO. Notably, increased levels of FTO have been shown to mitigate As-induced damage, while silencing FTO worsens the harmful effects.^103^ Further studies reveal that FTO protects against arsenite-induced oxidative stress and cognitive deficits by modulating the m^6^A modification of activating transcription factor 3 (ATF3) mRNA. Specifically, exposure to As decreases neuronal levels of FTO, leading to increased methylation of ATF3. This methylation promotes the degradation of ATF3 through recognition by YTHDC1, which exacerbates oxidative stress and neuronal injury.^104^

### Aluminum

Aluminum (Al) has been shown to have persistent neurotoxic effects, particularly during early developmental stages. Exposure to Al during the perinatal period disrupts neuronal development and significantly alters the levels of m^6^A modification.^105^ In mice exposed to Al, hippocampal neurons demonstrate functional impairments, which are characterized by a reduced expression of METTL3 and decreased levels of m^6^A modifications.^106^ Additionally, Al exposure leads to a reduction in FTO expression in the hippocampus, resulting in increased methylation of BDNF mRNA and decreased protein expression. This cascade of effects contributes to oxidative stress and apoptosis in the neurons.^107^

### Cobalt

Cobalt (Co) has been shown to disrupt sensory and memory functions within the CNS and is associated with neurodegenerative diseases. Studies involving Co-exposed mice and cellular models have observed alterations in m^6^A modification levels and regulatory proteins. Co exposure leads to the overproduction of ROS, which suppresses the expression of FTO, causes G1/S cell cycle arrest, and increases the levels of cleaved caspases 1, 3, and 9, ultimately accelerating apoptosis.^108^ Additionally, Co exposure impairs autophagy, resulting in the accumulation of the tau protein. The reduction in FTO expression due to Co exposure increases the methylation of tuberous sclerosis complex 1 (TSC1) mRNA, leading to elevated TSC1 levels. The TSC1-TSC2 complex then inhibits the mammalian target of the rapamycin complex, which induces autophagy.^109^ Beyond FTO, ALKBH5 also plays a significant role in mediating Co-induced neurotoxicity. Both clinical and *in vitro* studies indicate that exposure to Co decreases ALKBH5 levels. Interestingly, overexpression of ALKBH5 can restore cell viability and reduce apoptosis.^110^ Conversely, down-regulation of ALKBH5 exacerbates ferroptosis by affecting the regulation of heme oxygenase-1, leading to further neuronal damage.^111^

## Targeting m^6^A modification for neurological disease therapy

As previously mentioned, m^6^A modification plays a crucial role in regulating the differentiation of neural cells. Dysregulation of this process has been linked to the development of various neurological disorders. Therefore, targeting m^6^A modification could be a promising therapeutic strategy for these conditions.

### Involvement of m^6^A modification in damage repair

In conditions such as ischemic stroke and spinal cord injury, assessing the severity of neuronal damage and progress in recovery is crucial for determining prognosis. Research has shown that the up-regulation of FTO can reduce the size of infarcts in ischemic stroke, alleviate oxidative stress, decrease neuronal apoptosis, and accelerate the restoration of motor, cognitive, and other functions. FTO achieves this by lowering the methylation levels of Nrf2 mRNA, which hinders its degradation and protects cells from damage caused by oxidative stress.^90^^,^^112^^,^^113^ Additionally, targeting Drp1 through FTO has been found to alleviate mitochondrial dysfunction and oxidative stress following ischemic stroke, thereby restoring neuronal function.^114^ Additionally, the suppression of METTL3 has been demonstrated to enhance neuronal survival and alleviate cell cycle arrest after ischemia–reperfusion injury.^115^ In the case of spinal cord injury, both METTL3 and METTL14 have been recognized as essential factors, with multiple studies indicating that inhibiting either of these enzymes can significantly enhance neuronal regeneration after injury.^116^, ^117^, ^118^

Furthermore, m^6^A modification has been identified as a crucial factor in the process of axon regeneration. Overexpression of METTL3 has been shown to greatly improve the regenerative potential of peripheral nerves.^119^ After an injury to the peripheral sciatic nerve, numerous repair-related genes are activated, and proteins are synthesized in the dorsal root ganglion to support axon repair. The absence of METTL14 or YTHDF1 inhibits the synthesis of axon-associated proteins, such as ATF3, which leads to a decrease in effective axon regeneration. In contrast, the presence of ALBKH5 promotes the up-regulation of Lpin2, thereby hindering neuronal regrowth. In the CNS, the deletion of ALBKH5 improves the survival of retinal ganglion cells and facilitates axon regeneration following optic nerve damage. However, suppression of METTL14 impairs axon regeneration induced by Pten deficiency in retinal ganglion cells.^120^^,^^121^ In cerebellar granule cells, YTHDF1 and YTHDF2 play significant roles in regulating the translation of Dvl1 and the stability of Wnt5a, respectively. Both proteins are coordinated with the Wnt5a pathway to negatively influence the axon growth of granule cells.^31^ Previous studies have demonstrated that m^6^A can regulate axonal growth. Furthermore, the deletion of YTHDF1 in hippocampal neurons results in structural and functional abnormalities in excitatory synapses.^78^ In AD patients, reduced m^6^A modification levels are associated with decreased synthesis of synaptic proteins, such as CAMKII and GLUA1, leading to diminished synaptic plasticity.^83^ In the local synaptic protein synthesis pathway composed of presynaptic terminal-postsynaptic terminal-glial cells, the levels of m^6^A modification affect the transcription rate of local proteins and thus affect the synthesis of local synaptic proteins.^122^ It may be a useful way to induce or inhibit the development of axons and synaptic connections by regulating the abundance of m^6^A, so as to achieve the purpose of repairing the damage of nervous system.

### The way to regulate m^6^A modification

Abnormalities in m^6^A-related enzymatic proteins can lead to various pathological conditions. These issues may be improved by modulating the expression of m^6^A modifications through external pathways. Exosomes serve as promising delivery vectors due to their low immunogenicity and ability to cross the blood–brain barrier. In a study involving a PD model in mice, researchers administered exosomes loaded with specific FTO siRNA to the striatal region. This treatment suppressed α-syn expression and significantly enhanced the survival of dopaminergic neurons.^65^ Additionally, the introduction of exosomes containing FTO into LPS-induced microglia promoted the polarization of these cells toward the M2 phenotype and reduced the inflammatory response associated with ischemic stroke.^63^ Exosomes sourced from mesenchymal stem cells have been shown to up-regulate FTO expression, ameliorate mitochondrial dysfunction, and mitigate neuronal injury in ischemic stroke.^114^ Furthermore, researchers have successfully reinstated METTL3 expression in autistic mice through the administration of lentiviruses carrying METTL3. This intervention resulted in improved apoptosis of hippocampal neurons and a partial restoration of social communication behaviors and mobility.^123^ Moreover, exosomes derived from NSCs containing YBX1 interact with IGF2BP1 to suppress NLRP3 vesicle activation during ischemic stroke, thereby reducing neuronal apoptosis.^124^ In addition to exosome use, another strategy involves direct genetic modification. Specifically, targeted manipulation of m^6^A modification levels can be achieved through the CRISPR-dCas13 system. However, this approach is currently limited to animal model development and requires significant efforts before it can be implemented clinically.^125^, ^126^, ^127^

**Figure 3:**
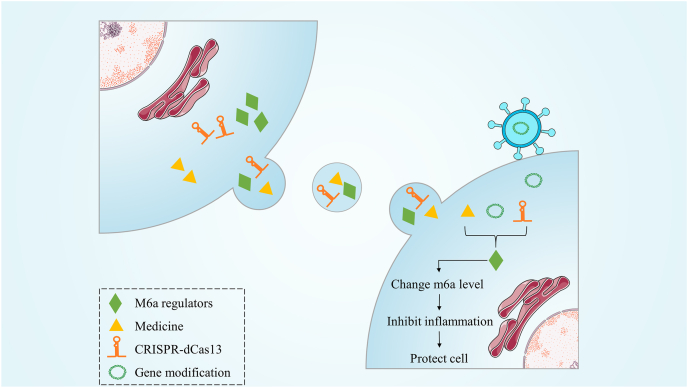
The way to regulate m^6^A modification. Through exosomal or lentiviral delivery, substances can be transferred to target cells, altering their m6A levels and thereby achieving therapeutic effects.

In addition to directly delivering m^6^A modifications or modifying genes related to m^6^A, alternative substances can be used to indirectly influence the activity of m^6^A-related enzymatic proteins. This can lead to changes in m^6^A modification levels in the body, which may achieve therapeutic objectives.^128^ For example, inhibitors of FTO, such as rhein and meclofenamic acid, have been studied. Research suggests that meclofenamic acid can inhibit the progression of glioblastoma by suppressing FTO activity.^129^ Additionally, compound 21 has been shown to enhance METTL3 expression, helping to alleviate the neuronal damage induced by isoflurane.^130^ Therefore, targeting m^6^A modifications might provide a novel therapeutic strategy for CNS disorders. Exosomes can serve as effective carriers for delivering m^6^A modifications (Fig. 3).

## Conclusion

In summary, m^6^A modification is essential for the development of the nervous system. Its regulatory effects can sometimes seem contradictory. For example, the knockout of FTO can either inhibit or promote the proliferation and differentiation of NSCs; however, the resulting neurons often exhibit abnormal morphology and impaired function. This bidirectional regulation is influenced by various factors, including species, cell type, and the cellular state, indicating that m6A-mediated regulation in the nervous system is a complex and temporally specific process, with individual factors acting both independently and synergistically. This bidirectional regulation is also evident in conditions such as AD, PD, and toxin-induced neurodegenerative disorders. In certain contexts, m^6^A can have neuroprotective effects, while in others, it may contribute to disease progression. The specific role of m^6^A varies depending on the underlying cause of the disease model, as well as the site and type of injured cell. Thus, m6A represents a promising avenue for exploring the mechanisms of toxin-induced neurological diseases, and further research will deepen our understanding of neurodegenerative pathology.

## CRediT authorship contribution statement

**Zhou She:** Writing – original draft. **Peng Huang:** Writing – review & editing. **Senlin Luo:** Writing – review & editing. **Lu Zhang:** Writing – review & editing. **Hong Peng:** Writing – review & editing. **Yufen Tang:** Writing – review & editing. **Yuqiong Chen:** Writing – review & editing. **Jinwen Luo:** Writing – review & editing. **Wangxin Duan:** Writing – review & editing. **Lingjuan Liu:** Writing – review & editing. **Liqun Liu:** Writing – review & editing.

## Funding

This project was supported by the 10.13039/501100001809National Natural Science Foundation of China (No. 81873762), the Science and Technology Department Program of Hunan Province, China (No. 2023SK4018), the 10.13039/501100004735Natural Science Foundation of Hunan Province, China (No. 2024JJ8249, 2024JJ8235), the Fundamental Research Funds for the Center Universities of Central South University (China) (No. 2024ZZTS0161).

## Conflict of interests

There is not conflict of interests.
